# Anatomical study of the brachial plexus in human fetuses and its relation with neonatal upper limb paralysis

**DOI:** 10.31744/einstein_journal/2020AO5051

**Published:** 2020-01-22

**Authors:** Marcelo Rodrigues da Cunha, Amanda Aparecida Magnusson Dias, Jacqueline Mendes de Brito, Cristiane da Silva Cruz, Samantha Ketelyn Silva

**Affiliations:** 1 Faculdade de Medicina de Jundiaí JundiaíSP Brazil Faculdade de Medicina de Jundiaí, Jundiaí, SP, Brazil.; 2 Centro Universitário Padre Anchieta JundiaíSP Brazil Centro Universitário Padre Anchieta, Jundiaí, SP, Brazil.

**Keywords:** Brachial plexus/anatomy & histology, Fetus/abnormalities, Paralysis

## Abstract

**Objective:**

To study the anatomy of the brachial plexus in fetuses and to evaluate differences in morphology during evolution, or to find anatomical situations that can be identified as the cause of obstetric paralysis.

**Methods:**

Nine fetuses (12 to 30 weeks of gestation) stored in formalin were used. The supraclavicular and infraclavicular parts of the brachial plexus were dissected.

**Results:**

In its early course, the brachial plexus had a cord-like shape when it passed through the scalene hiatus. Origin of the phrenic nerve in the brachial plexus was observed in only one fetus. In the deep infraclavicular and retropectoralis minor spaces, the nerve fibers of the brachial plexus were distributed in the axilla and medial bicipital groove, where they formed the nerve endings.

**Conclusion:**

The brachial plexus of human fetuses presents variations and relations with anatomical structures that must be considered during clinical and surgical procedures for neonatal paralysis of the upper limbs.

## INTRODUCTION

The brachial plexus is composed of the anterior branches of the spinal nerves from C5 to T1, and their nerve roots are responsible for the sensory and motor innervation of the upper limbs. However, the topographic location of this plexus makes it susceptible to trauma due to its anatomical proximity to oscillating neck and shoulder structures, and to absence of muscle and bone protection.^1^

In newborns, most neonatal brachial plexus palsy (NBPP) occurs due to the elongation of the brachial plexus during delivery; however, there are documented cases of this injury without traction of the fetal head, and the term “obstetric paralysis” has been opposed by several authors due to, among other factors, advances in modern obstetrics.^2 - 4^ There are other associated risk factors, such as dystocia, in which the fetus’s shoulder is trapped against the maternal pubic symphysis, which can create tension along the upper part of the fetal brachial plexus.^5^ The newborn’s weight may also be related to this dystocia.^4^ Other causes of NBPP may be involved, such as gestational or pre-gestational diabetes, macrosomic fetus, pelvic deliveries with cervical hyperextension of the newborn, idiopathic and obesity.^6^ Perinatal asphyxia causes hypotonia, which predisposes plexus injuries from stretching.^7^ It is still unclear if the use of forceps is a risk and if Cesarean sections (C-sections) are safer, although this mode of delivery does not completely remove the risk of NBPP.^4 , 8 , 9^

Brachial plexus injury causes sensorimotor loss and deformities, such as contractures, due to bone and joint alterations in the upper limb, in cases of incomplete recovery. Nonetheless, in most cases, the patients recover spontaneously.^9 - 11^ Obstetric brachial paralysis (OBP) occurs in approximately 1 to 3:1,000 liveborn,^1^ and the classification parameter is defined according to the affected nerve roots. The injuries that affect the upper trunk of the plexus (C5 - C6 and, sometimes, C7) are called Erb-Duchenne palsy, and those affecting the lower trunk (C8 – T1) are called Klumpke palsy. Moreover, there is complete paralysis, in which there is avulsion of all nerve roots.^12 - 14^

The diagnosis is based on physical examination through the passive and painful movement of the affected limb, absence of active movement, flaccid paralysis, loss of the flexor pattern, and trophic changes of the skin.^15 , 16^ Electroneuromyography conducted after the 10^th^ day and before the 60^th^ day of life can also help with the injury’s prognosis.^1 , 11^

Therefore, it is important clinicians be familiar with the anatomy of fetuses and newborns so they can choose the best treatment and tests in cases of NBPP. Moreover, there is little information about the anatomy of the brachial plexus in fetuses, and it is crucial to have this kind of information to better understand the etiologies involved in brachial plexopathies, and surgical procedures in the shoulder girdle region in children.

## OBJECTIVE

To study the anatomy of the brachial plexus in fetuses and evaluate the differences in morphology during evolution, or to find anatomical situations that may be determined to be the cause of obstetric palsies.

## METHODS

This is a descriptive study using nine formaldehyde-preserved fetus cadavers (five of them male), between 12 and 30 weeks of gestation, belonging to the anatomy laboratory of the *Centro Universitário Padre Anchieta* (UNIANCHIETA). Of the five male fetuses, three were between 12 and 20 weeks, and the other two between 28 and 30 weeks. Two of the female fetuses were between 12 and 20 weeks, and the other two were between 28 and 30 weeks. This study was approved by the Research Ethics Committee from UNIANCHIETA, CAAE: 58070716.0.0000.5386, protocol no. 1.674.106. The anatomical dissection of the supra- and infraclavicular regions was done with surgical instruments to cut and detach the skin, subcutaneous tissue, fasciae, and muscles, thus exposing the trunks and fascicles of the brachial plexus and their relation to the adjacent bone structures and soft tissues. To standardize the procedure, the dissection of the brachial plexus of all fetuses was performed by one single researcher. This study was conducted during the second semester of 2016.

## RESULTS

**Figure 1:**
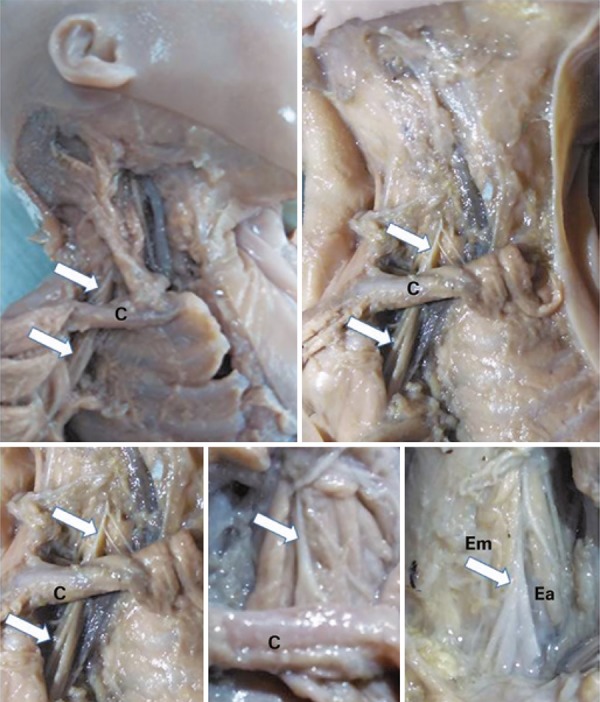
Macroscopic images of dissection of the brachial plexus of human fetus cadavers. Note the brachial plexus (arrows) in the regions superior and inferior to the clavicle, and the relation to the hiatus between the anterior and middle scalene muscles

Source: Anatomy laboratory of the *Centro Universitário Padre Anchieta* .

**Figure 2:**
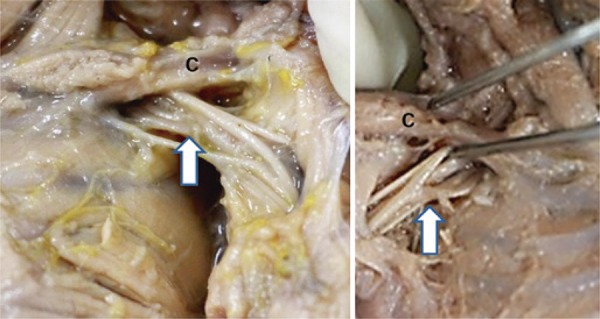
Macroscopic image of the infraclavicular region of human fetus cadavers. Note the brachial plexus (arrow) going inferiorly through the middle third of the clavicle and the distribution of its terminal branches in the inferior axillary region

Source: Anatomy laboratory of the *Centro Universitário Padre Anchieta* .

In the supraclavicular region of all fetuses studied, the brachial plexus emerging as a united single cord was found to be in deep in relation to the sternocleidomastoid muscle and in the hiatus, between the anterior and middle scalene muscles. In only one fetus the origin of the phrenic nerve was observed through the brachial and not the cervical plexus. The brachial plexus cord branched out into the upper, middle, and lower trunks going posteriorly through the middle third of the clavicle. In the infraclavicular region, the brachial plexus was medial to the shoulder joint and anterolateral to the subclavian vein. The fascicles of the brachial plexus were located posteriorly to the pectoralis minor muscle, and were distributed to form the terminal nerves in the axillary and scapular regions and in the medial bicipital groove, such as the musculocutaneous, radial, ulnar, axillary and median nerves, which were the most visible during dissection ( Figures 1 and 2 ). In the fetuses between 12 and 20 weeks of gestation, we saw that the brachial plexus was shaped as a single cord, and the division into trunks only occurred in the region posterior to the clavicle. However, in the fetuses between 28 and 30 weeks of gestation, the division into trunks occurred in the supraclavicular region, showing the evolution of the brachial plexus ramifications.

C: clavicle; Ea: anterior scalene muscle; Em: middle scalene muscle.

## DISCUSSION

The brachial plexus is vulnerable in both the peri- and post-natal periods. However, there is little information in the literature about its development in human fetuses, and further knowledge is required to better understand the mechanisms involved in its injuries. Understanding the morphological variations of the brachial plexus is crucial in neurology and traumatology, and these alterations can explain unexpected clinical signs and symptoms – that is why some studies show the importance of studying the brachial plexus in human fetuses.^17 - 19^ According to Fazan et al., the brachial plexus presents important relations to other local anatomical structures, thus predisposing anatomical variations with clinical and surgical repercussions.^20^ Milanov et al., highlighted these important relations when they conclude that revascularized nerves of human fetuses can also be used as grafts to replace post-traumatic defects in peripheral nerves through microsurgical techniques.^21^

The classic anatomical descriptions state the brachial plexus passing between the anterior and middle scalene muscles, with its trunks of supraclavicular location and fascicles in an infraclavicular position. The upper trunk of the brachial plexus is often formed by the cervical nerves C5 and C6; the middle trunk is formed by the C7; and the lower trunk by the C8 and T1.^22 , 23^ Similarly, our study also noted the relation of the brachial plexus in the hiatus of the scalene in fetuses. Fodor et al., observed anatomical anomalies in the thoracocervicoaxillary region, such as hypertrophy of the anterior scalene muscle in fetuses, and concluded that the compression of brachial plexus cords in this region may cause thoracic outlet syndrome.^19^ However, other structures may be related, as described by Fazan et al., when they noticed that the phrenic nerve was entirely originated from the plexus in 20% of the studied fetuses.^20^ Similarly, our study observed this variation in only one fetus, but this is noteworthy information because an injury in the brachial plexus in the neck could result in an inexplicable paralysis of the diaphragm muscle.

Another result observed in this study was the natural tension of the brachial plexus along its trajectory, which supports the theory that the trauma mechanism of OBP occurs during excessive traction of the cephalic pole, which further stretches the nerve structures.^24 , 25^ Moreover, there was no anatomical variation of the brachial plexus between the antimers, which is different from what was found by Woźniak et al., who identified alterations more often on the left side of the studied fetuses, which can make them predisposed to injuries from excessive stretching.^17 , 18^ In contrast to previous findings, Uysal et al., studied 200 brachial plexus from fetuses, and observed that the most frequent morphologic variations occurred on the right side and in female fetuses. They also noticed differences in the formation of the trunks, where the upper trunk can be formed with the contribution of the cervical nerve C4, and the lower trunk through the thoracic nerve T2. In some cases, the upper and lower trunks were missing, and some nerves were connected, as occurred between the median and the musculocutaneous nerves.^26^ In comparison to these results, our study showed that the brachial plexus fascicles were already formed, but they were grouped and the terminal branches were distributed to the axilla and the medial bicipital groove, with no connection between the nerves that could characterize anatomic variation. However, the fragility of the anatomical structures of the axillary regions and the prolonged preservation in formaldehyde of the fetus cadavers limited a more detailed study of the muscle and cutaneous branches of each terminal component of the brachial plexus. Therefore, further studies are warranted to continue the research about brachial plexus paralysis in the embryologic phase and its clinical application.

Woźniak et al., studied 220 the brachial plexus of 110 human fetuses aged between 14 to 32 weeks of fetal life, and observed important variations of the cord with a higher frequency in the anterior division of the middle trunk, as noted in 63 (28.63%) of the cases.^17 , 18^ Therefore, understanding the development of the brachial plexus is paramount for clinical diagnosis and pediatric surgeries of the neck, axilla and arms in newborns or during first childhood when there is injury to the brachial plexus.^27^

## CONCLUSION

The brachial plexus of human fetuses presents topographic relations with the scalene muscles. The studied specimens showed us that the brachial plexus has a different morphology before the 20^th^ week. After 28 weeks, there is no difference between its morphology and syntopy from those of adult plexus. Obstetric palsies can be explained by fragility of the structures.
