# Supplementary material for: Anatomical study of the brachial plexus in human fetuses and its relation with neonatal upper limb paralysis
Source: Einstein (Sao Paulo). 2020 Jan 22;18:eAO5051. doi: 10.31744/einstein_journal/2020AO5051 (PMC6980293; doi:10.31744/einstein_journal/2020AO5051)
Supplement: Translation

# Translation: Estudo anatômico do plexo braquial de fetos humanos e sua relação com paralisias neonatais do membro superior

**Authors:** Marcelo Rodrigues da Cunha, Amanda Aparecida Magnusson Dias, Jacqueline Mendes de Brito, Cristiane da Silva Cruz, Samantha Ketelyn Silva

**Affiliations:** 1 Brasil Faculdade de Medicina de Jundiaí, Jundiaí, SP, Brasil.; 2 Brasil Centro Universitário Padre Anchieta, Jundiaí, SP, Brasil.

**Keywords:** Plexo braquial/anatomia & histologia, Feto/anormalidades, Paralisia

## Abstract

**Objetivo:**

Estudar a anatomia do plexo braquial em fetos e avaliar diferenças de morfologia durante a evolução, ou encontrar situações anatômicas que possam ser apontadas como causa de paralisias obstétricas.

**Métodos:**

Foram utilizados nove fetos formolizados entre 12 a 30 semanas de gestação e submetidos à dissecação supra e infraclavicular do plexo braquial.

**Resultados:**

O plexo braquial inicialmente tem formato de cordão durante sua passagem pelo hiato dos escalenos e em apenas um feto foi observada a origem do nervo frênico por meio do plexo braquial. Na região infraclavicular profunda e retropeitoral menor, os fascículos do plexo braquial se distribuíam na axila e sulco bicipital medial para a formação dos nervos terminais.

**Conclusão:**

O plexo braquial de fetos humanos apresenta variações e relações com estruturas anatômicas que devem ser consideradas durante os procedimentos clínicos e cirúrgicos das paralisias neonatais do membro superior.

## INTRODUÇÃO

O plexo braquial é composto pelos ramos anteriores dos nervos espinhais de C5 a T1, cujas raízes nervosas são responsáveis pela inervação sensitiva e motora do membro superior. Entretanto, a localização topográfica deste plexo torna-o suscetível a traumas devido a sua proximidade anatômica com estruturas oscilantes do pescoço e ombro, além da falta de proteção muscular e óssea.^1^

Em recém-nascido, a maioria das paralisias neonatal do plexo braquial (PNPB) ocorre pelo alongamento do plexo braquial durante o parto, porém há casos documentados da ocorrência desta lesão sem a tração da cabeça do feto, e o termo “paralisia obstétrica” tem sido condenado por vários autores, até mesmo pelo avanço da obstetrícia moderna.^2 - 4^ Assim, outros fatores de riscos estão associados, como a distocia, em que o ombro do feto fica preso na sínfise púbica materna, podendo tensionar a parte superior do plexo braquial fetal.^5^ O peso do recém-nascido também pode estar relacionado com essa distocia.^4^ Outras causas das PNPB podem estar envolvidas, como diabetes gestacional ou pré-gestacional, fetos macrossômicos, partos pélvicos com hiperextensão cervical do recém-nascido, idiopática e obesidade.^6^ A asfixia perinatal provoca hipotonia, que predispõe às lesões do plexo por estiramento.^7^ Ainda há dúvidas se o uso de fórceps constitui um risco, e as cesarianas são mais seguras, porém não eliminam totalmente o risco da PNPB.^4 , 8 , 9^

A lesão do plexo braquial provoca perda sensório-motora e deformidades, como as contraturas, devido às alterações ósseas e articulares do membro superior em casos de recuperação incompleta, entretanto a maioria se recupera espontaneamente.^9 - 11^ A incidência da paralisia braquial obstétrica (PBO) está na média de 1 a 3:1.000 nascidos vivos,^1^ e o parâmetro de classificação é definido de acordo com as raízes nervosas agredidas. As lesões que afetam o tronco superior do plexo (C5-C6, às vezes C7) são chamadas de paralisia de Erb-Duchenne e as que afetam o tronco inferior (C8-T1) são chamadas de paralisia de Klumpke. Além disso, há a paralisia completa, na qual todas as raízes nervosas são avulsionadas.^12 - 14^

O diagnóstico é baseado em exame físico da movimentação passiva e dolorosa do membro afetado, ausência da movimentação ativa, paralisia flácida, perda do padrão flexor e alterações tróficas da pele.^15 , 16^ A eletroneuromiografia realizada após o décimo dia e antes dos 60 dias de vida também pode ajudar no prognóstico da lesão.^1 , 11^

Desta maneira, é importante que o clínico conheça a anatomia do feto e do recém-nascido para que possa escolher o melhor tratamento e exames em uma situação de PNPB. Além disso, são escassas as informações referentes à anatomia do plexo braquial em fetos, as quais se fazem essenciais para o melhor conhecimento das etiologias envolvidas nas plexopatias braquiais, assim como nos procedimentos cirúrgicos na região da cintura escapular em crianças.

## OBJETIVO

Estudar a anatomia do plexo braquial em fetos e avaliar as diferenças de morfologia durante a evolução, ou encontrar situações anatômicas que possam ser apontadas como causa de paralisias obstétricas.

## MÉTODOS

Estudo descritivo com a utilização de nove cadáveres de fetos formolizados (cinco do sexo masculino), entre 12 a 30 semanas de gestação, pertencentes ao Laboratório de Anatomia do Centro Universitário Padre Anchieta (UNIANCHIETA). Dos cinco fetos do sexo masculino, três tinham entre 12 a 20 semanas e os outros dois entre 28 a 30 semanas. Quanto aos femininos, dois tinham entre 12 a 20 semanas e os outros dois entre 28 a 30 semanas. A pesquisa foi aprovada pelo Comitê de Ética da UNIANCHIETA, com CAAE: 58070716.0.0000.5386 e parecer 1.674.106. A dissecação anatômica das regiões supra e infraclavicular foi realizada com materiais cirúrgicos para seccionar e rebater pele, tela subcutânea, fáscias e músculos, possibilitando a exposição dos troncos e fascículos do plexo braquial e sua relação com estruturas ósseas e tecidos moles adjacentes. Para a padronização, a dissecação do plexo braquial de todos os fetos foi realizada por um único pesquisador. Esta pesquisa foi realizada durante o segundo semestre de 2016.

## RESULTADOS

Na região supraclavicular de todos os fetos estudados, o plexo braquial em forma de tronco único localizou-se profundamente em relação ao músculo esternocleidomastóideo e no hiato, entre os músculos escalenos anterior e médio. Em apenas um feto, foi observada a origem do nervo frênico por meio do plexo braquial e não do cervical. O cordão do plexo braquial se ramificava em troncos superior, médio e inferior, ao passar posteriormente pelo terço médio da clavícula. Na região infraclavicular, o plexo braquial apresentava-se medialmente à articulação do ombro e anterolateralmente à veia subclávia. Os fascículos do plexo braquial localizavam-se posteriormente ao músculo peitoral menor e se distribuíam para a formação dos nervos terminais na região axilar, escapular e bicipital medial, como o musculocutâneo, radial, ulnar, axilar e mediano − sendo estes os mais visíveis durante a dissecção ( Figuras 1 e 2 ). Nos fetos de 12 a 20 semanas gestacionais, notou-se que o plexo braquial tinha formato de cordão único, e a divisão em troncos somente ocorreu na região posterior à clavícula. Entretanto, naqueles com 28 a 30 semanas de período fetal, a divisão em troncos ocorreu na região supraclavicular, demonstrando a evolução das ramificações do plexo braquial.

**Figura 1:** Imagens macroscópicas da dissecação do plexo braquial de cadáveres de fetos humanos. Note o plexo braquial (setas) nas regiões superior e inferior à clavícula, bem como a relação com o hiato entre os músculos escalenos anterior e médio

Fonte: Laboratório de Anatomia do Centro Universitário Padre Anchieta.

C: clavícula; Ea: músculo escaleno anterior; Em: músculo médio.

**Figura 2:** Imagem macroscópica da região infraclavicular de cadáveres de fetos humanos. Observe o plexo braquial (seta) passando inferiormente ao terço médio da clavícula e a distribuição de seus ramos terminais na região axilar inferior

Fonte: Laboratório de Anatomia do Centro Universitário Padre Anchieta.

## DISCUSSÃO

O plexo braquial é vulnerável em ambos os períodos perinatal e pós-natal, porém são escassas na literatura as informações sobre seu desenvolvimento em fetos humanos, para que fossem melhor entendidos os mecanismos envolvidos nas lesões. Considerando que o conhecimento das variações morfológicas do plexo braquial é importante na neurologia e na traumatologia, e que essas alterações podem ainda explicar sinais e sintomas clínicos inesperados, algumas pesquisas demonstram a importância do estudo do plexo braquial de fetos humanos.^17 - 19^ De acordo com Fazan et al., o plexo braquial apresenta relações importantes com outras estruturas anatômicas locais, predispondo ao aparecimento de variações anatômicas com repercussão clínica e cirúrgica.^20^ Milanov et al., destacam esta importância ao concluírem que nervos revascularizados do feto humano também podem ser usados como enxertos para a substituição de defeitos pós-traumáticos nos nervos periféricos, por meio de técnicas microcirúrgicas.^21^

As descrições anatômicas clássicas referem que o plexo braquial tem trajetória entre os músculos escalenos anterior e médio, com seus troncos de localização supraclavicular e fascículos em posição infraclavicular. O tronco superior do plexo braquial é frequentemente formado pelos nervos cervicais C5 e C6; o tronco médio por C7; e o tronco inferior por C8 e T1.^22 , 23^ De forma semelhante, também foi observada nesta pesquisa em fetos a relação do plexo braquial no hiato dos escalenos. Fodor et al., observaram anomalias anatômicas da região toraco-cervicoaxilar como hipertrofia do músculo escaleno anterior de fetos e concluíram que a compressão dos cordões do plexo braquial neste local pode provocar a síndrome do desfiladeiro torácico.^19^ Porém, outras estruturas não comuns também podem estar relacionadas como descrito por Fazan et al., ao notarem que o nervo frênico se originou inteiramente do plexo em 20% dos fetos estudados.^20^ De forma semelhante, nesta pesquisa foi constatada essa variação em apenas um feto, mas destaca-se essa informação, pois uma lesão no plexo braquial no pescoço poderia resultar em paralisia inexplicável do músculo diafragma.

Outro resultado observado nesta pesquisa foi a tensão natural do plexo braquial ao longo de seu trajeto, sustentando a teoria de que o mecanismo de trauma da PBO se dá durante a tração excessiva do polo cefálico, que provoca ainda mais o estiramento das estruturas nervosas.^24 , 25^ Além disso, não houve variação anatômica do plexo braquial entre os antímeros, diferentemente do encontrado por Woźniak et al., que identificaram alterações mais no lado esquerdo dos fetos estudados, podendo predispor às lesões por alongamento excessivo.^17 , 18^ Contrariando os achados anteriores, Uysal et al., estudaram 200 plexos braquiais de fetos e observaram que as variações morfológicas de maior frequência foram no lado direito e em fetos femininos. Notaram também diferenças na formação dos troncos, sendo que o superior pode ser formado com a contribuição do nervo cervical C4 e o inferior pelo torácico T2. Em alguns casos, eram inexistentes os troncos superior e inferior, e alguns nervos se conectavam, como ocorreu entre o mediano e o musculocutâneo.^26^ Comparando a estes resultados, notou-se, nesta pesquisa, que os fascículos do plexo braquial já estavam formados, porém agrupados e com distribuição dos ramos terminais para a axila e o sulco bicipital medial, sem conexão entre nervos que pudesse caracterizar variação anatômica. Entretanto, a fragilidade das estruturas anatômicas da região axilar e a formolização por longo tempo dos fetos cadavéricos limitaram um estudo mais detalhado dos ramos musculares e cutâneos de cada componente terminal do plexo braquial. Assim, outros estudos são importantes para a continuação das pesquisas do plexo braquial na fase embriológica e sua aplicação clínica.

Woźniak et al., estudaram 220 plexos braquiais de 110 fetos humamos com idades entre 14 a 32 semanas de vida fetal e observaram importantes variações do cordão com maior frequência na divisão anterior do tronco médio, conforme notado em 63 (28,63%) dos casos.^17 , 18^ Desta maneira, o conhecimento do desenvolvimento do plexo braquial é importante no diagnóstico clínico e nas cirurgias pediátricas do pescoço, axila e braço do recém-nascido, ou na primeira infância que apresenta alguma lesão do plexo braquial.^27^

## CONCLUSÃO

O plexo braquial de fetos humanos apresenta relações topográficas com os músculos escalenos. Os espécimes estudados nos permitem dizer que o plexo braquial possui morfologia diferente antes das 20 semanas. Após as 28 semanas, não há diferenças entre sua morfologia e sintopia com o plexo de adultos. As paralisias obstétricas podem ser explicadas pela delicadeza das estruturas.
